# Theoretical Analysis of Thermophysical Properties of 3D Carbon/Epoxy Braided Composites with Varying Temperature

**DOI:** 10.3390/polym16081166

**Published:** 2024-04-21

**Authors:** Li-Li Jiang, Zhen-Guo Li, Dong-Ye Wang, Jun-Jun Zhai, Xiang-Xia Kong

**Affiliations:** 1 College of Civil Engineering and Architecture, Xiamen City University, Xiamen 361008, China; jianglili@xmcu.edu.cn (L.-L.J.); lizhenguo@xmcu.edu.cn (Z.-G.L.); wangdy2269@outlook.com (D.-Y.W.); 2 College of Aeronautics and Astronautics, North China Institute of Aerospace Engineering, Langfang 065000, China; 3 Hebei Key Laboratory of Trans-Media Aerial Underwater Vehicle, North China Institute of Aerospace Engineering, Langfang 065000, China; 4 Department of Material Engineering, North China Institute of Aerospace Engineering, Langfang 065000, China; kongxx@nciae.edu.cn

**Keywords:** 3D braided composites, helix geometry unit cell, thermophysical properties, temperature

## Abstract

A three-dimensional helix geometry unit cell is established to simulate the complex spatial configuration of 3D braided composites. Initially, different types of yarn factors, such as yarn path, cross-sectional shape, properties, and braid direction, are explained. Then, the multiphase finite element method is used to develop a new theoretical calculation procedure based on the unit cell for predicting the impacts of environmental temperature on the thermophysical properties of 3D four-direction carbon/epoxy braided composites. The changing rule and distribution characteristics of the thermophysical properties for 3D four-direction carbon/epoxy braided composites are obtained at temperatures ranging from room temperature to 200 °C. The influences of environmental temperature on the coefficients of thermal expansion (CTE) and the coefficients of thermal conduction (CTC) are evaluated, by which some important conclusions are drawn. A comparison is conducted between theoretical and experimental results, revealing that variations in temperature exert a notable influence on the thermophysical characteristics of 3D four-directional carbon/epoxy braided composites. The theoretical calculation procedure is an effective tool for the mechanical property analysis of composite materials with complex geometries.

## 1. Introduction

Three-dimensional braided composites have been widely used in aerospace, automobile, and marine fields owing to the outstanding mechanical properties of high through-thickness strength and impact resistance over traditional laminated composites. In order to make full use of the advantages of 3D braided composites, it was important to clarify the mechanical behaviors of composites in different environments. Many researchers [1,2,3,4,5,6,7,8,9,10,11] have focused their efforts on the complex geometries and mechanical properties of 3D braided composites at room temperature. The influence of temperature on the thermal properties of fiber-reinforced composites has so far been a concern of some researchers. Mohajerjasbi [12] reported the CTE of three-dimensional braided composites using the finite element-based method. Yao et al. [13] studied the thermal expansion behavior and analyzed the anisotropy of the thermal response of the 3D braided structure by experiments. Wang et al. [14] predicted the thermoelastic properties of 3D braided composites and the numerical results were verified by experiments. Liang et al. [15] studied the effects of microcrack density on the effective thermal expansion coefficients of the material. Xia et al. [16] considered the meso-structure of 3D braided composites and calculated the global effective thermal conductivity coefficients and CTE. Gowayed et al. [17] and Schuster et al. [18] investigated the thermal conductivity of 3D fiber-reinforced composites by experiment. Liu et al. [19] and Li et al. [20] reported the thermal conductivity of braided composites based on different microstructure models. Cheng et al. [21] determined the thermal property of fiber composites by numerical methods and experiments. Lu [22] calculated the elastic constants and thermophysical properties of 3D full five-directional braided composites. Jiang and Wang et al. [23,24] predicted the effective coefficients of thermal expansion and interfacial thermal stress of 3D braided composites, and they evaluated how the braiding angle and fiber volume fraction impact the effective CTE, interfacial stress, and distribution. Tranchard et al. [25] measured the thermal physical properties, such as thermal decomposition, specific heat capacity, and thermal conductivity, of the carbon-reinforced epoxy composite laminate using different innovative characterized methods. Gou et al. [26] calculated the effective thermal conductivities of 3D braided composites based on the multi-size unit cells. Yu et al. [27] improved the through-thickness thermal conductivity of carbon/epoxy laminated composites and investigated the effective thermal conductivity of the composite panel. Dong et al. [28] characterized and analyzed the thermal conductivity of 3D braided composites in different directions by the finite element method and experiments. Wei et al. [29] predicted the effective thermal conductivities of 3D braided carbon/carbon composites considering randomly distributed void defects. Li et al. [30] studied the bending performance of braided/epoxy resin composites at different temperatures and heating time, respectively. The damage modes and failure mechanisms of the material were revealed. Zuo et al. [31] produced 3D five-directional braided carbon/epoxy composites and investigated the compression mechanical response and failure mechanisms of braided composites at elevated temperatures. Jiang et al. [32] explored the compressive mechanical behavior of braided composites and analyzed the failure mechanism. Zhai [33] developed a finite element simulation framework to describe the thermoviscoelastic behavior of braided composites. Hu [34] used computed tomography and the finite element method to characterize the relationship between the internal damage distribution of 3D braided carbon fiber/epoxy composites under multiple-transverse-impact loading, ambient temperature, and microstructure. Arbaouiet et al. [35] researched the thermomechanical properties of 3D braided composite materials. Fang et al. [36] accomplished the high-precision computation of effective thermal conductivity based upon a geometry model of parameterized modified 3D braided composites. Wu et al. [37] reported the low-velocity impact behaviors and the degradation mechanism of fiber-reinforced composites in different directions after thermo-oxidative ageing.

The aerospace craft, automobile, and marine working environments are highly complex and harsh, involving aerodynamic heating during rocket flight and heat generated by instrument work, which all create a hostile thermal environment that is accompanied by thermal conduction. This will cause changes in the stress and strain of structural components, resulting in thermal stress, which in turn affects the normal work of the structural components and the mechanical properties of the materials. Therefore, 3D braided composites are required to have a high level of environmental adaptability and be able to maintain high structural stability in severe environmental changes. The ability of 3D braided composites, used as structural materials, to accommodate temperature variations in their operational environment is critically dependent on their thermophysical properties. The thermal conductivity of composites determines the exchange of thermal energy with the surroundings and their own temperature dynamics. Their thermal expansion properties, on the other hand, determine the stability of their structural dimensions, directly influencing stress distribution, thermal shock resistance, and the integrity of component interfaces. It directly affects the heat impact resistance, heat conduction, and heat dissipation performance of components, which has a significant impact on the lifespan and operational ranges of components. The current research works are of great significance for further exploration of the thermomechanical behavior of braided composites. However, the varying temperature with time will affect the thermomechanical behavior of fiber-reinforced composites and cannot be avoided. Research about the effect of varying temperature on the thermomechanical properties of braided composites is very limited at present. In our previous work, a novel multiphase FEM [38,39,40,41] was proposed to analyze the mechanical behaviors of 3D braided composites. In this paper, the thermophysical properties are mainly studied for 3D carbon/epoxy braided composites at temperatures ranging from room temperature to 200 °C. The thermophysical properties are first compared with experimental data and is then followed by predicting the influence of environmental temperature on the thermophysical properties.

## 2. Geometry of Yarn Architectures

**Figure 1:**
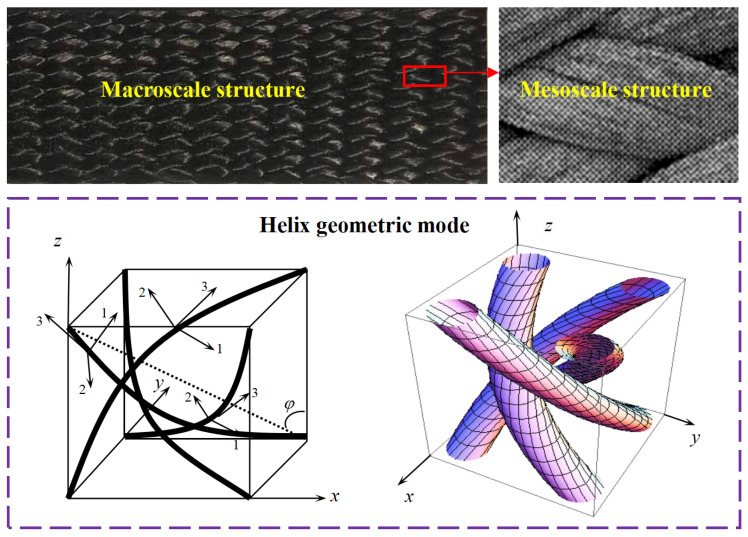
Helix geometry unit cell of 3D four-dimensional braided composites.

To precisely quantify the variation rule of mechanical properties for 3D braided composites, a modified helix geometry model was presented in our previous work [39]. Because of the complex geometric structure of 3D braided composites, the cross-section of the yarn is assumed to be an ellipse and the curved yarn paths are employed to overcome the cross in the center of the geometry model. All the yarns are unidirectional composites with transversely isotropic elastic properties. As shown in Figure 1, in order to determine the geometric configuration of the curved yarns, the global coordinate system (X-Y-Z) is employed, where the Z-axis is the braid direction and X-Y is the transverse direction. The local coordinate system (1-2-3) is determined along the geometry configuration of yarns to describe the yarn material parameters, where the 3-axis is the longitudinal orientation along the yarn and 1-2 is the transverse orientation.

## 3. Finite Element Formulation

**Figure 2:**
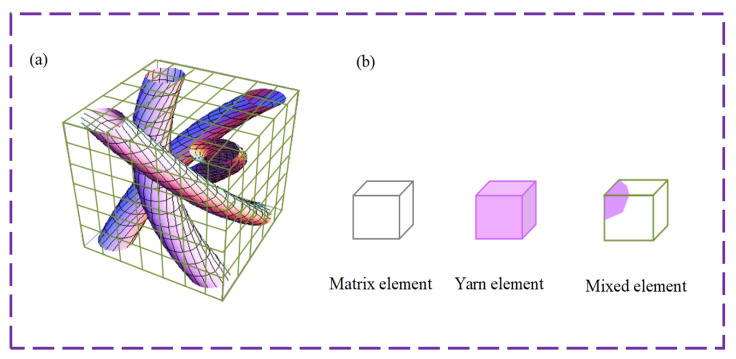
Microscopic RVE: (**a**) RVE; (**b**) three kinds of elements. Reproduced with permission from reference [42].

For the conventional numerical analysis method, the braiding yarn and matrix are modeled discretely. This is more tedious and time consuming due to the complex discretization. In order to simplify the analysis of mechanical properties of 3D fiber-reinforced composites, the multiphase finite element method is adopted. The micro-scale RVE is divided into numerous isoparametric rectangular sub-cells including three types of elements as shown in Figure 2. The yarn elements include only the yarns, the matrix elements include only the resin, and the mixed elements are composed of yarn and resin. This numerical discretization method is brief and is easy to implement. Subsequently, the finite element analysis programs are developed to predict the thermophysical properties of 3D four-direction braided composites under different temperatures.

### 3.1. CTE of 3D Braided Composites

Let {*F_T_*}, [*K*], and {*δ_T_*} denote the equivalent thermal nodal force, the total equivalent stiffness matrix, and the nodal degree of freedom related to the materials, respectively. The finite element equation of the thermal load problem can be stated as

(1) $$\left\{F_{T}\right\}=\left[K\right]\left\{\delta_{T}\right\}$$

The stiffness matrix [*K*] can be calculated according to the method described in Ref [39]. The sum of equivalent thermal nodal forces of all sub-cells is the equivalent thermal nodal force {*F_T_*}:

(2) $$\left\{F_{T}\right\}=\sum_{e=1}^{M+N+L}\left\{F_{T}^{i}\right\}\;\left(i=Y,M,Mix\right)$$

where *Y*, *M*, and *Mix* denote the yarn/resin/mixed sub-cells, respectively. *M*, *N,* and *L* are the number of three kinds of sub-cells, respectively. $\left\{F_{T}\right\}$ depends on the temperature boundary condition. The equivalent thermal nodal force of each sub-cell can be obtained by

(3) $$\left\{F_{T}^{i}\right\}=\int_{V}{\left[B\right]}^{T}\left[D_{i}\right]\left\{\varepsilon_{i}\right\}dV=\int_{-1}^{1}\int_{-1}^{1}\int_{-1}^{1}{\left[B\right]}^{T}\left[D_{i}\right]\left\{\varepsilon_{i}\right\}det\left[J\right]d\xi d\eta d\zeta \;$$

where [*B*] represents the strain displacement matrix and [*J*] represents the Jacobian matrix, and [*D_i_*] and {ε*_i_*} denote the property matrix and the thermal strain matrix of materials, respectively. 

In this work, to obtain the equivalent thermal nodal force of each sub-cell, the formula of 27 Gauss-integration-point quadrature is applied to calculate the numerical integration of all sub-cells. Therefore, Equation (3) can be obtained as follows:

(4) $$\left\{F_{T}^{i}\right\}=\sum_{r=1}^{3}\sum_{s=1}^{3}\sum_{t=1}^{3}W_{r}\bar{W_{s}}\tilde{W_{t}}{\left({\left[B\right]}^{T}\left[D_{i}\right]\left\{\varepsilon_{i}\right\}det\left[J\right]\right)}_{\xi =\xi_{r},\eta =\eta_{s},\zeta =\zeta_{t}}$$

where *ξ_r_*, *η_s_*, and *ζ_t_* are the Gauss integral points and $W_{r}$, ${\bar{W}}_{s}$, and ${\tilde{W}}_{t}$ are the relevant weight coefficients. The material property matrix [*D_i_*] can be obtained according to the method described in Ref. [35]. The thermal strain {ε*_i_*} can be calculated according to the position of Gauss integral points. If the integral point is within the yarn volume, {ε*_i_*} is replaced by {ε*_Y_*}; otherwise, it is replaced by {ε*_M_*}.

**Figure 3:**
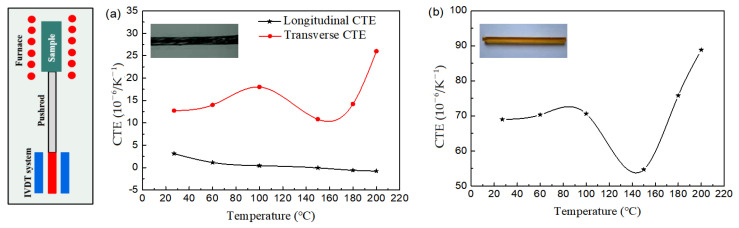
CTE variation with environmental temperature of braiding yarn and matrix: (**a**) carbon fiber yarn; (**b**) matrix.

The 3D carbon/epoxy braided composite consists of carbon fiber yarns and resin. The matrix elements are isotropic and the carbon fiber yarns are regarded as the homogeneous material and transversely isotropic. When there is only a uniform temperature change and no external mechanical loads, the thermal strain of the matrix and carbon fiber yarn in the global coordinate system can be represented as follows:

(5) $$\left\{\varepsilon_{M}\right\}={\left[\begin{array}{ccc} \alpha_{m}\Delta T & \alpha_{m}\Delta T & \alpha_{m}\Delta T \end{array}\;\begin{array}{ccc} 0 & 0 & 0 \end{array}\right]}^{T}$$

(6) $$\left\{\varepsilon_{Y}\right\}={\left[T_{R}\right]}^{T}\left\{\varepsilon_{Y}^{\prime }\right\}={\left[T_{R}\right]}^{T}{\left[\begin{array}{ccc} \alpha_{C}\Delta T & \alpha_{C}\Delta T & \alpha_{L}\Delta T \end{array}\;\begin{array}{ccc} 0 & 0 & 0 \end{array}\right]}^{T}$$

in which *α_m_* is the CTE of the matrix and Δ*T* represents the temperature rise value from some reference temperature without thermal strain. [*T_R_*] represents the transformation matrix, $\left\{\varepsilon_{Y}^{\prime }\right\}$ represents the thermal strain of the braiding yarn in the material coordinate system, and *α_c_* and *α_L_* are the transverse and longitudinal CTEs of the braiding yarn, respectively. The CTEs of the braiding yarn and matrix are measured by a Netzsch DIL 402 Expedis at temperatures ranging from 25 °C to 200 °C and under a heating rate of 3 K/min. The testing principle is illustrated in Figure 3, where LVDT stands for Linear Variable Differential Transformer, which is connected to a pushrod. By contacting the sample, the change in sample length is detected, from which the thermal expansion spectrum of the sample can be obtained. Table 1 shows the elastic properties of the used composites. The dimension of each sample used in this test is 25 × 6 × 6 mm^3^ and made by the Institute of Composite Materials of Tianjin Polytechnic University. Figure 3 presents the CTEs of the braiding yarn and matrix at different temperatures.

The nodal degree of freedom {*δ_T_*} related to the materials can be calculated by solving Equation (1) combined with the boundary conditions. Then, the thermal strain of each element can be obtained by

(7) $$\left\{\varepsilon_{T}\right\}=\left[B\right]\left\{\delta_{T}\right\}$$

In this study, the homogenization approach is applied to calculate the effective CTEs of 3D four-direction carbon/epoxy braided composites. The materials are regarded as homogeneous on the macrolevel. Under an arbitrary temperature change Δ*T*, the average normal strain of the materials in any direction can be represented as

(8) $$\left\{{\bar{\varepsilon }}_{jT}\right\}=\frac{1}{V}\int_{V}\left\{\varepsilon_{jT}\right\}dV\;\left(j=x,y,z\right)$$

The effective CTE of 3D braided composites can then be rewritten as

(9) $$\left\{{\bar{\alpha }}_{jT}\right\}=\frac{1}{\Delta T}\left\{{\bar{\varepsilon }}_{jT}\right\}$$

### 3.2. CTC of 3D Braided Composites

Let $\left\{F_{c}\right\}$ denote the equivalent nodal heat flux, $\left[K_{C}\right]$ represent the total thermal stiffness matrix, and $\left\{T\right\}$ signify the nodal temperature of the materials. The finite element equation of the steady-state heat conduction problem can be rewritten as

(10) $$\left\{F_{c}\right\}=\left[K_{c}\right]\left\{T\right\}$$

The sum of the equivalent nodal heat flux of all sub-cells is the equivalent nodal heat flux $\left\{F_{c}\right\}$*,* as Equation (11) shows:

(11) $$\left\{F_{c}\right\}=\sum_{e=1}^{M+N+L}{\left\{F_{c}\right\}}^{e}$$

where $\left\{F_{c}\right\}$ is subject to the boundary condition and the heat flux imposed on elements. The total thermal stiffness matrix can be expressed as

(12) $$\left[K_{c}\right]=\sum_{e=1}^{M}{\left[K_{c}^{Y}\right]}^{e}+\sum_{e=1}^{N}{\left[K_{c}^{M}\right]}^{e}+\sum_{e=1}^{L}{\left[K_{c}^{Mix}\right]}^{e}\;$$

where ${\left[K_{c}^{Y}\right]}^{e}$, ${\left[K_{c}^{M}\right]}^{e}$, and ${\left[K_{c}^{Mix}\right]}^{e}$ represent the thermal stiffness matrix of the yarn/matrix/mixed element yarn element, respectively. 

The thermal stiffness matrix of these three kinds of elements can be calculated by

(13) $${\;\left[K_{c}^{i}\right]}^{e}=\int {\left[B\right]}^{T}\left[k_{c}^{i}\right]\left[B\right]dV=\int_{-1}^{1}\int_{-1}^{1}\int_{-1}^{1}{\left[B\right]}^{T}\left[k_{c}^{i}\right]\left[B\right]det\left[J\right]d\xi d\eta d\zeta \;$$

in which [*B*] represents the strain displacement matrix, $\left[k_{c}^{i}\right]$ represents the thermal conductivity matrix, and [*J*] represents the Jacobian matrix. 

In this study, to obtain the thermal stiffness matrix of each sub-cell, the formula of 27 Gauss-integration-point quadrature is applied to calculate the numerical integration of all sub-cells. Therefore, Equation (13) can be stated as

(14) $${\;\left[K_{c}^{i}\right]}^{e}=\sum_{r=1}^{3}\sum_{s=1}^{3}\sum_{t=1}^{3}W_{r}\bar{W_{s}}\tilde{W_{t}}{\left({\left[B\right]}^{T}\left[k_{c}^{i}\right]\left[B\right]det\left[J\right]\right)}_{\xi =\xi_{r},\eta =\eta_{s},\zeta =\zeta_{t}}$$

**Figure 4:**
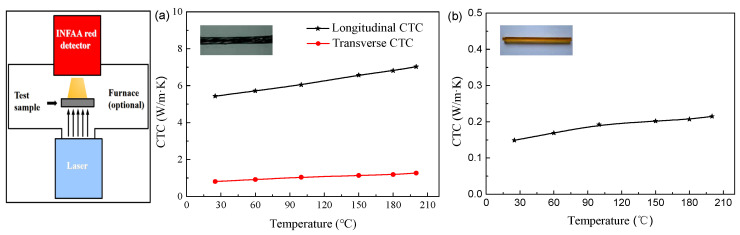
CTC variation with environmental temperature of braiding yarn and matrix: (**a**) carbon fiber yarn; (**b**) matrix.

The thermal conductivity tensors of the matrix and braiding yarn in the global coordinate system can be, respectively, written as

(15) $$\left[k_{c}^{M}\right]=\left[\begin{array}{ccc} \lambda_{m} & 0 & 0\\ 0 & \lambda_{m} & 0\\ 0 & 0 & \lambda_{m} \end{array}\right]$$

(16) $$\left[k_{c}^{Y}\right]={\left[T_{G}\right]}^{-1}\left[k_{c}^{Y\prime }\right]\left[T_{G}\right]$$

where $\lambda_{m}$ is the CTC of the matrix, $\left[T_{G}\right]$ signifies the transformation matrix, and $\left[k_{c}^{Y’}\right]$ represents the thermal conductivity tensor of the carbon fiber yarn in the local coordinate system and can be stated as

(17) $$\left[k_{c}^{Y’}\right]=\left[\begin{array}{ccc} \lambda_{T} & 0 & 0\\ 0 & \lambda_{T} & 0\\ 0 & 0 & \lambda_{L} \end{array}\right]$$

where $\lambda_{T}$ and $\lambda_{L}$ are the transverse and longitudinal CTCs of the braiding yarn, respectively. The CTCs of the braiding yarn and matrix are measured by a Netzsch LFA467 HyperFlash HT at temperatures ranging from 25 °C to 200 °C. The basic principle of the test is shown in Figure 4. At a certain set temperature, a laser source emits a pulse of light instantaneously, which uniformly illuminates the lower surface of the sample. After the surface absorbs the light energy, the temperature rises instantaneously, and at the hot end, it propagates the energy as one-dimensional heat conduction. Thus, the CTC of the sample at the set temperature can be measured. The dimension of each sample used in this test is 6 × 6 × 1 mm and made by the Institute of Composite Materials of Tianjin Polytechnic University. Figure 4 presents the CTCs of the braiding yarn and matrix at different temperatures.

The heat conductivity tensor $\left[k_{c}^{Mix}\right]$ of the mixed element depends on the coordinates of the Gauss integration points. When the Gauss integration point is located in the yarn region, the heat conductivity tensor $\left[k_{c}^{Y}\right]$ is selected; otherwise, the heat conductivity tensor $\left[k_{c}^{M}\right]$ is selected. 

According to the law of heat conduction of anisotropic solids, the equivalent thermal conductivity coefficients ${\bar{\lambda }}_{j}$ in any direction can be obtained by

(18) $$\left\{{\bar{\lambda }}_{j}\right\}=-\frac{{\bar{q}}_{j}}{{\bar{\vartheta }}_{j}}$$

where ${\bar{\vartheta }}_{j}$ signifies the total average temperature gradient, and ${\bar{q}}_{j}$ denotes the total average heat flux rate and can be represented as follows:

(19) $$\left\{{\bar{\vartheta }}_{j}\right\}=\frac{1}{V}∭{\left\{\vartheta_{j}\right\}}^{e}dv\;$$

(20) $$\left\{{\bar{q}}_{j}\right\}=\frac{1}{V}∭{\left\{q_{j}\right\}}^{e}dv\;$$

where ${\left\{\vartheta_{j}\right\}}^{e}$ is the temperature gradient and ${\left\{q_{j}\right\}}^{e}$ is the heat flux rate of an element, which can be, respectively, determined by

(21) $${\left\{\vartheta_{i}\right\}}^{e}={\left[\begin{array}{ccc} \frac{\partial T}{\partial x} & \frac{\partial T}{\partial y} & \frac{\partial T}{\partial z} \end{array}\right]}^{T}=\left[B\right]{\left\{T\right\}}^{e}$$

(22) $${\left\{q_{i}\right\}}^{e}=-\left[k_{c}^{i}\right]{\left\{\vartheta_{i}\right\}}^{e}$$

in which ${\left\{T\right\}}^{e}$ denotes the nodal temperature of an element and can be determined by solving Equation (10).

## 4. Numerical Calculation and Discussions

According to the above finite element method, the Fortran codes are written to predict, respectively, the CTEs and CTCs of 3D carbon/epoxy braided composites under different temperatures. In the numerical calculation, three kinds of braiding angle (25°, 35°, and 45°) and a constant-fiber-volume fraction of 58% are taken into account. The mechanical properties of the fiber and resin are listed in Table 1.

### 4.1. Coefficients of Thermal Expansion

**Figure 5:**
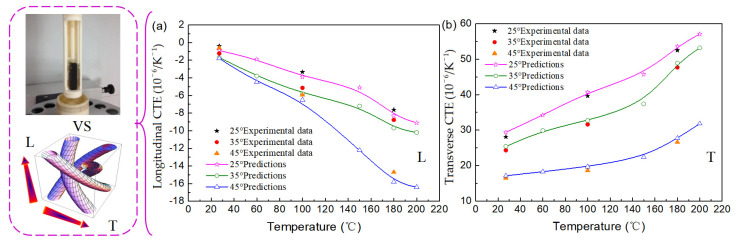
CTE variation with environmental temperature of 3D braided composites: (**a**) longitudinal CTE; (**b**) transverse CTE.

The CTEs of 3D four-direction carbon/epoxy braided composites are calculated at temperatures ranging from 25 °C to 200 °C and the results are shown in Figure 5. The left side of Figure 5 shows the experimental determination for the CTEs of 3D braided composites and the numerical calculation model using the multiphase finite element method. It is observed that the longitudinal CTEs are all negative or zero and decrease with increasing temperature. Due to the negative expansion along the fiber axial direction decreasing with temperature (Figure 3a), the longitudinal CTEs of 3D four-direction carbon/epoxy braided composites are negative. At 200 °C, the longitudinal CTEs are, respectively, 10.1, 6.0, and 9.1 times that at 25 °C for the composites with 25°, 35°, and 45° braiding angles, respectively. 

As shown in Figure 5b, the transverse CTEs increase with the increase in temperature, but at a lower rate until the temperature increases above 150 °C. It can be seen (Figure 3b) that the matrix has a significant impact, due to the CTE of the matrix growing rapidly at temperatures greater than 150 °C. At 200 °C, the transverse CTEs are, respectively, 1.9, 2.1, and 1.9 times those at 25 °C for the composites with 25°, 35°, and 45° braiding angles. 

Meanwhile, it is found that there is a significant difference in the CTEs in the respective longitudinal and transverse directions, which shows that the thermal expansion of 3D carbon/epoxy braided composites obviously represents anisotropy, especially at high temperature. The influence of environmental temperature on the CTEs of 3D carbon/epoxy braided composites is significant. It is not easy to maintain the original shape of the 3D four-direction carbon/epoxy braided composites, and they are prone to longitudinal shrinkage and transverse expansion. This effect becomes more pronounced as the temperature increases. However, the small braiding angle composites have better dimensional stability, thermal shock resistance, and thermal impact resistance compared to large-braiding-angle composites. Therefore, in engineering, it is important to select reasonable braiding parameters to minimize the thermal expansion coefficient in a specific direction, even approaching zero. This allows the composites to maintain dimensional stability under large temperature differences, thus meeting the requirements for dimensional stability and durability in fields such as aerospace, precision instruments, and other related industries. These prediction results are also in accordance with the experimental data reported in ref. [40].

### 4.2. Coefficients of Thermal Conductivity

**Figure 6:**
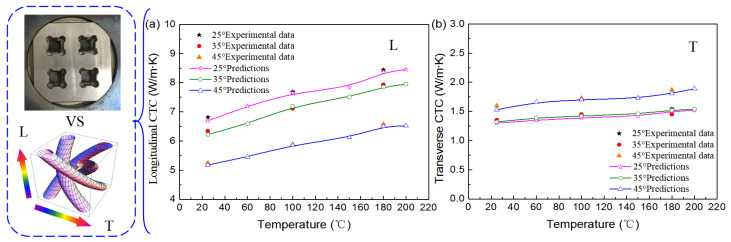
CTC variation with environmental temperature of 3D braided composites: (**a**) longitudinal CTC; (**b**) transverse CTC.

Figure 6 illustrates the CTCs of 3D four-direction carbon/epoxy braided composites at temperatures ranging from 25 °C to 200 °C. The left side of Figure 6 shows the experimental determination for the CTCs of 3D braided composites and the numerical calculation model using the multiphase finite element method. It can be seen that the longitudinal and transverse CTCs all increase linearly with the increase in temperature. This is mainly attributed to the CTCs of the carbon fiber yarn and matrix all increasing with temperature (Figure 4). With increasing temperature from 25 °C to 200 °C, the longitudinal CTCs increase by 26.3%, 27.8%, and 25.9%, and the transverse CTCs increase by 16.9%, 16.7%, and 23.5% for the composites with 25°, 35°, and 45° braiding angles, respectively. The longitudinal thermal conductivity decreases continuously with the increase in braiding angle, and the variation in the braiding angle has an obvious effect on the longitudinal thermal conductivity of 3D braided composites. This is mainly due to the tendency of 3D four-direction carbon/epoxy braided composites with small braiding angles to be oriented towards unidirectional fiber-reinforced-resin-based composites, and the fiber bundles have a higher thermal conductivity along the fiber direction, which increases the longitudinal thermal conductivity of 3D braided composites. As shown in Figure 6b, the changes in temperature and braiding angle have a minimal impact on the transverse CTC of 3D braided composites. The longitudinal and transverse CTC of 3D carbon/epoxy braided composites differ significantly, indicating that the thermal conductivity behavior of 3D braided composites exhibits obvious anisotropic characteristics. Moreover, the smaller the braiding angle, the more pronounced the anisotropic characteristics; at the same braiding angle, the higher the temperature, the more pronounced the anisotropic characteristics.

The temperature has notable influence on the CTCs of 3D four-direction carbon/epoxy braided composites. The theoretical calculation values of CTCs are compared with the experimental data in the relevant literature [40] and the predictions are in good agreement with the corresponding measurements.

## 5. Conclusions

A new theoretical approach to using the multiphase finite element method to model the thermophysical properties of 3D four-direction carbon/epoxy braided composites has been suggested. The impacts of environmental temperature on the CTEs and CTCs of 3D four-direction carbon/epoxy braided composites are evaluated using the present approach. The conclusions are as follows:

(1) The environmental temperature has significant impact on the thermal expansion properties of 3D four-direction carbon/epoxy braided composites. As the temperature rises, the components are prone to longitudinal shrinkage and lateral expansion. The higher the temperature, the more pronounced the deformation effects. With increasing temperature from room temperature to 200 °C, the longitudinal CTEs are all negative or zero and decrease, while the transverse CTEs increase, but at a lower rate until the temperature increases above 150 °C.
(2) The longitudinal and transverse CTCs all increase linearly with the increase in temperature. As the temperature rises, the heat exchange of 3D braided composites with the surroundings decreases, and its temperature change trend slows down.
(3) The braiding angle has notable influence on the thermophysical properties of 3D four-direction carbon/epoxy braided composites. With increasing temperature, the size stability of the braided composites with small braiding angles is superior to that of the braided composites with large braiding angles, while the thermal insulation performance of the braided composites with large braiding angles is better than that of the braided composites with small braiding angles.
(4) Meanwhile, the anisotropy of the thermophysical properties of 3D four-direction carbon/epoxy braided composites is clearly observed. And as the braiding angle decreases, the anisotropic characteristics become more distinct; at the same braiding angle, the higher the temperature, the more pronounced the anisotropic characteristics.
(5) The outcomes achieved with the current approach align with the experimental findings, which confirms the precision and viability of the method in forecasting the thermophysical characteristics of 3D four-direction carbon/epoxy braided composites. The approach, capable of integration into commercial finite element software, proves to be an effective tool for designing and optimizing the structure of heterogeneous materials exhibiting anisotropic properties or complex geometries.

## Figures and Tables

**Table 1:** Mechanical properties of carbon fiber (CF) and expoxy resin (EP).

Component Materials	E_33f_/GPa	E_22f_/GPa	G_32f_/GPa	G_12f_/GPa	E_m_/GPa	γ_32f_	γ_m_
Carbon fiber	215.6	17.21	12.92	9.3	—	0.3	—
Matrix	—	—	—	—	3.45	—	0.35

## Data Availability

Data are contained within the article.
